# The impact of household digital transformation on household energy efficiency: Empirical evidence from Chinese households

**DOI:** 10.1371/journal.pone.0315372

**Published:** 2024-12-09

**Authors:** Suxu Lin, Lin He, Xin Lin, Weicheng Li

**Affiliations:** 1 School of Management, Guangdong Polytechnic Normal University, Guangzhou, Guangdong, China; 2 School of Finance and Economics, Guangdong Polytechnic Normal University, Guangzhou, Guangdong, China; China University of Geosciences, CHINA

## Abstract

Carbon emissions from household consumption are an important part of global energy consumption, and household digital transformation is vital for realizing green and low−carbon development. Using data from the 2019 China Household Finance Survey, this study empirically examines the effect of household digital transformation on household energy efficiency. The results show that household digital transformation significantly improves household energy efficiency across all quantiles. This effect varies by consumption type, with the most pronounced improvements in food, housing, and other consumption categories. The impact is stronger for households with elderly members. Per capita household income and education level serve as important mediating and moderating mechanisms, respectively. Unlike previous macro-level studies, this research provides micro-evidence on the impact of household digital transformation on energy efficiency, explores the underlying mechanisms through income and education effects, and examines heterogeneous impacts across different consumption types and household characteristics. These findings offer important policy implications for leveraging digital transformation to enhance household energy efficiency and promote sustainable development.

## 1 Introduction

Rapid climate change is the most urgent crisis for humanity, and the large amount of energy consumption is the main cause of this change. Gases emitted from energy consumption is estimated to account for about 60% of global greenhouse gas (GHG) emissions each year [1]. The Global Climate Status Report 2021 published by the World Meteorological Organization (WMO) shows that the last seven years have been the hottest on record. As such, GHG emissions must be urgently reduced, and sustainable development must subsequently be realized. However, the rapid development of China’s economy and the steady growth of household consumption in recent years have led to the increase in energy consumption and carbon dioxide emissions [2]. China has become the world’s largest energy consumer and CO_2_ emitter [3]. Notably, the household is the basic unit of residential energy consumption and carbon emissions [4]. In China, the household sector accounts for more than 40% of the country’s total CO_2_ emissions [5]. In the United States, residential energy use accounts for about 20% of GHG emissions [6]. Household energy consumption is one of the main reasons for the increase in global energy demand and carbon emissions, and the household sector plays an indispensable role in meeting China’s sustainable development needs [7]. Optimizing household consumption is necessary for promoting energy use and driving carbon emissions [8, 9].

Digital technologies offer potential solutions for reducing energy consumption and carbon emissions. The United Nations has proposed in “Transforming our World: The 2030 Agenda for Sustainable Development” to double the global rate of energy efficiency improvement by 2030; moreover, improving energy efficiency is critical to achieving long-term climate goals. Green consumption concepts and lifestyle changes are recognized as necessary for long−term reductions in GHG emissions [10]. As the world’s largest carbon emitter and a global manufacturing hub, China’s efforts in this area are particularly important. The rapid development of China’s digital economy provides significant opportunities for addressing climate change. In recent years, the digital economy has become a major force for economic development, and the level of development of China’s digital economy has increased year by year. In the future, China’s economy will rely increasingly on domestic demand and, consequently, final consumption. The household sector plays a key role in the overall goal of China’s energy revolution as well as in meeting the demand for long−term sustainability [11]. Hence, forming a sustainable household consumption pattern has become the most urgent challenge to be solved nowadays.

This study investigates the impact of household digital transformation on household energy efficiency. Specifically, we use data from the 2019 China Household Finance Survey (CHFS) [12] to conduct empirical analysis. We employ OLS regression and instrumental variable method for baseline regression and endogeneity treatment. The main finding is that household digital transformation can significantly improve household energy efficiency. Further analysis shows this enhancement effect exists across households at different quantiles, has heterogeneous impacts on energy efficiency of different consumption types like food and housing, and has a greater impact on households with elderly members. We also reveal that per capita household income and education level are important mediating and moderating mechanisms. Robustness tests and heterogeneity analysis are conducted to verify the reliability of conclusions. This research provides empirical evidence for household digital transformation to improve energy efficiency, which has policy implications for achieving sustainable development.

The contributions of this paper are as follows: Firstly, unlike previous research that primarily focused on macro-level analyses [17–19], we provide micro-evidence on the impact of digital transformation on energy efficiency at the household level. Secondly, we delve deeper into the mechanisms underlying the relationship between digital transformation and energy efficiency, particularly examining the mediating roles of household income and education levels—an aspect often overlooked in existing research [42, 43]. Finally, we conduct a detailed heterogeneity analysis, exploring how the effects of digital transformation vary across different consumption types and household characteristics, thus offering insights for targeted policy formulation.

The remainder of this study is presented below. Section 2 briefly reviews the literature on digital transformation and energy efficiency. Section 3 describes the data sources and the econometric methodology. Section 4 gives the empirical results. Section 5 presents the conclusions and related policy implications.

## 2 Background and hypothesis

As the construction of China’s information infrastructure continues to advance, the Internet penetration rate and the size of Internet users continue to grow steadily, and people are beginning to build their lives around digital technology. These innovations are continuously penetrating all aspects of family life and production, with Internet−based digital technologies gradually becoming crucial in daily activities. Individuals’ social behaviors, lifestyles, and consumption patterns are influenced by digital technology, and people are building their families’ lives around the possibilities it offers. This process is regarded as household digital transformation. At the same time, such transformation has been accompanied by an increase in the consumption of goods and services, such as digital finance, online health care, and online education. With the development of digital technology, telecommuting has freed people from workspace constraints.

However, this shift has led to increased household energy consumption and carbon emissions. For example, households purchasing computers, communicating with colleagues, and providing heating and lighting consume energy and emit carbon. As such, household digital transformation will inevitably change the level and structure of household consumption. Digital applications have gradually covered the basic needs of households. The development of digital technology is closely related to the scale of energy consumption, energy structure, and energy intensity [13], and the significant increase in energy consumption has become an important support for rapid economic development [14].

Energy efficiency is the key to solving environmental problems and developing a green economy [15]. Energy efficiency usually refers to the ratio of expected energy consumption to actual energy consumption, which includes not only the technical dimension but also the economic and social dimensions [16]. Digital technology is permeating human life, and improving energy efficiency is a key way to mitigate global warming. Established studies provide macro evidence of the impact of digital transformation on energy efficiency mainly at the regional level. Wu et al. [17] examined the impact of the digital economy on energy efficiency in Chinese cities, and the results of the study showed that the digital economy would improve the energy efficiency of cities in the initial stage. Wang and Shao et al. [18] also confirmed the positive effect of the digital economy on energy efficiency. They also found that this facilitating effect gradually increases as the level of economic development increases. The findings of Song et al. [19] are also consistent; it indicated that digitization can promote better energy efficiency. However, this improvement is heterogeneous and depends on several factors, such as resource endowment, city size, and geographical location. Lin and Huang [20] demonstrated through cross−country panel data that digitization can improve energy efficiency and save energy when the digitization level reaches 0.4324. In addition, Zhao et al. [21] further confirmed that the Internet and mobile devices can improve energy efficiency and reduce energy structure and intensity [22]. Unlike the concept of energy efficiency in macro studies, the household energy efficiency described in this paper refers to the ratio of household carbon emissions to indirect energy consumption to reflect the carbon dioxide emissions per unit of indirect energy consumption [23].

The widespread application of digital technology has promoted the transformation of traditional household activities to digitalization, gradually realizing household digital transformation. Household digital transformation can be quantified by measuring the accessibility and usage of digital technologies, such as smartphone usage, computer ownership, mobile payment adoption, and online shopping behavior. These indicators, as suggested by previous studies [54, 55], reflect the level of digitalization within households. For example, smartphone and computer use enable digital interactions that can enhance energy efficiency, while mobile payments and online shopping can alter consumption patterns and reduce energy use. This approach aligns with findings at the macro level that digitalization improves energy efficiency [17, 18], making it relevant for household-level analysis. This transformation has changed the level and structure of household consumption and promoted the optimization of household appliances, personal environmental protection behaviors, and green lifestyles [24]. Moreover, the integration of the Internet with power−consuming devices, such as Internet of Things (IoT), and smart devices, can further improve household energy efficiency [25, 26]. Thus, carbon emissions and energy consumption are improved, and energy efficiency is promoted. Taken together, the impact of digitalization on energy efficiency is positive [27].

Existing studies have provided valuable insights into the relationship between digitalization and energy efficiency. However, several limitations in current research underscore the need for a more nuanced, micro-level examination:

1. While researchers have demonstrated that digital economy development can improve energy efficiency at macro levels [17–19], there is a gap in understanding how digital transformation affects energy efficiency at the household level [4, 8, 9].
2. Although some scholars have examined the impact of household consumption on energy use and carbon emissions [2, 8], the specific role of household digital transformation remains underexplored.
3. Previous research has investigated the effects of demographic factors on energy consumption [42, 43], but there is a lack of comprehensive analysis on the mechanisms through which household digital transformation influences energy efficiency. Additionally, the heterogeneous effects across different consumption types and household characteristics have not been adequately addressed. These limitations highlight the importance of a more detailed, household-level analysis of digital transformation’s impact on energy efficiency.

Therefore, we propose the following hypothesis:

Hypothesis 1: Household digital transformation can improve household energy efficiency.

The gap in consumption expenditure between different types of households is more evident, and the calculation of household energy efficiency is derived from household consumption values. Relevant studies consider eight types of consumption as the main source of household carbon emissions, including food, clothing, housing, transportation and communication, and medical treatment [28–30]. In contrast, the impact of digital economy exhibits heterogeneity on carbon emissions from eight categories of consumption, which significantly reduces the carbon emissions from the consumption of clothing, housing, transportation, and medicine [31]. Along with the development of digitalization, people are more likely to expand their online expenditures, thus reducing the associated carbon emissions. At the same time, population aging changes consumption patterns, thus affecting energy use as well as electricity consumption [32, 33]. When the number of offspring within the household gradually increases and the parents get older, household consumption gradually becomes more expensive in terms of spending on old−age care and education. Additionally, changes in the consumption structure occur, which may affect energy efficiency as well. Therefore, we believe that the structure of household members also affects the level and structure of household consumption expenditures, thus making differences in household energy efficiency.

In summary, we formulate Hypothesis 2:

Hypothesis 2a: Household digital transformation has a heterogeneous impact on energy efficiency across consumption types.
Hypothesis 2b: Household digital transformation has a heterogeneous impact on the energy efficiency of households with or without elderly.
Hypothesis 2c: Household digital transformation has a heterogeneous impact on the energy efficiency of households with or without minors.

In the digital age, stable economic growth and energy efficiency are important for achieving sustainable development [34]. Household digital transformation means that family members acquire additional digital skills and digital literacy. Firstly, advanced digital skills optimize household financial asset allocation by enhancing access to information and forming risk attitudes [35]. Secondly, the development of the digital economy and the acquisition of digital skills break through the limitations of people’s employment in terms of time and space. Moreover, they provide new opportunities to increase their income. Studies have shown that the development of digital economy promotes the use of digital technology, increases labor demand [36, 37], equalizes entrepreneurial opportunities for rural residents [38], raises the income level of residents [39], and narrows the residents’ income gap. Finally, the development of digital technology has reconfigured the way of human capital accumulation [40]. Households learn knowledge and technology on the Internet anytime and anywhere after digital transformation, which improves the human capital level of family members. The optimization of household asset allocation, the improvement of employment outcomes, and the enhancement of human capital accumulation effectively contribute to the increase of household income, which subsequently influences household consumption patterns. If the proportion of the consumption of high implied carbon intensity products changes, the associated energy efficiency will also change accordingly.

Thus, building on the previous section, we propose Hypothesis 3:

Hypothesis 3a: The household digital transformation is mainly through an increase in per capita household income, which in turn affects household energy efficiency.

Digital literacy varies significantly across social groups, and educational attainment is an important factor in accessing the digital dividend [41]. In addition to being related to digital literacy and competence, a person’s educational background is closely tied to environmental awareness, green consciousness, and attitudes toward ecological issues. Environmental awareness is the key to environmental governance and the development of consciousness to realize the harmonious coexistence of human beings with nature and the environment. In the process of receiving education, strengthening green awareness through environmental education can make citizens realize their own environmental responsibilities and obligations while improving their education level. in this process people can always remember the original intention and goal of environmental governance to realize the collective conscientiousness toward green development. The level of education has a significant effect on energy consumption [42], and the proportion of higher education is correlated with carbon dioxide emissions [43]. For instance, given that people with higher education are more aware of environmental protection [44], they are more inclined to use public transportation [45]. Studies have also shown that consumers’ education level has a significant moderating effect on the willingness to purchase new energy vehicles [46].

In summary, we propose Hypothesis 3b:

Education per household has a significant moderating effect on the impact of digital transformation on energy efficiency.

The United Nations 2030 Sustainable Development Goals were proposed to address the three dimensions of society, economy, and environment. Moreover, an increasing number of scholars are focusing on energy consumption research. Previous studies have shown that energy use and energy consumption are mainly influenced by the level of education [47], income [48, 49], population aging [32], access to credit [50], household size [51], and urbanization [52]. Other factors on energy use as well as energy consumption have been widely discussed. Currently, digital technologies are integrated into the daily lives of households, thus giving rise to new consumption patterns, providing novel consumption channels, and reducing spatial distances. Despite household energy accounting for a large share of the total energy demand, the impact of digital transformation on household energy efficiency is still under−researched and fails to provide highly comprehensive insights into energy efficiency and digital transformation.

## 3 Data and models

### 3.1 Data sources

The China Household Finance Survey (CHFS) is a comprehensive nationwide project collecting micro-level household financial information [12]. It employs a stratified, three-stage, PPS sampling method, ensuring low rejection rates and data closely aligned with census results, thus providing representative insights into Chinese household financial behaviors. The CHFS covers a wide range of household economic aspects, including housing assets, finances, liabilities, income, consumption, and demographic characteristics.

This study utilizes the full sample data from the 2019 CHFS, which encompasses 29 provinces, autonomous regions, and municipalities across China. By using the complete 2019 CHFS dataset, this research benefits from a nationally representative sample, enhancing the reliability and generalizability of our findings on household digital transformation and energy efficiency in China [12]. After excluding observations with missing data for key variables and evident outliers, our final sample consists of 12,485 households.

### 3.2 Benchmark regression model

To test the impact of household digital transformation on household energy efficiency, the following model is developed:

$${lnEFFI}_{i}=\alpha_{0}+ln{DIGIT}_{i}+\beta_{j}\sum X_{i}+{Province}_{i}+\varepsilon_{i}$$ (1)

In model (1), *lnEFFI*_*i*_ stands for household energy efficiency. *lnDIGIT*_*i*_ represents the digital transformation of households. *X*_*ij*_ is a series of control variables, including the age of the household head, gender of the household head, size of the household, proportion of the elderly population, and home ownership. *Province*_*i*_ is a province fixed effect. *ε*_*i*_ represents a randomized disturbance term.

### 3.3 Variable selection

#### 3.3.1 Household energy efficiency (EFFI)

Following Zhou et al. [50], household energy efficiency (EFFI) is defined as the ratio of household carbon emissions to indirect energy consumption. This indicator reflects the carbon dioxide emissions per unit of indirect energy consumption. The lower the value is, the higher the energy efficiency will be. Both household carbon emissions and indirect energy consumption are calculated using the Consumer Lifestyle Approach (CLA). This method maps household consumption to corresponding industry sectors, allowing for the estimation of energy use and carbon emissions associated with various types of household expenditure. Household CO_2_ emissions and indirect energy consumption are the products of carbon intensity, energy intensity, and household consumption expenditure calculated using the industry sector. To summarize, the current study refers to the energy and carbon intensities of consumer expenditures in all relevant sectors accounted for by the authors from 2005 to 2019, and Table 1 demonstrates the carbon and energy intensities corresponding to the eight categories of consumption in 2019.

**Table 1:** Energy intensity and carbon intensity conversion values (2019).

Consumption categories	Energy intensity	Carbon intensity
Food	7.18	63.92
Clothing	6.46	56.59
Resident	35.71	311.67
Equipment	2.05	18.41
Medical	4.02	35.69
Travel	1.67	14.20
Education	17.26	155.50
Other	2.23	19.05

We multiply the various types of household consumption and their corresponding energy intensity and carbon intensity obtained from the 2019 CHFS data. Accordingly, we calculate the carbon emissions and energy consumption of the eight types of household consumption in the 2019 CHFS. Finally, household energy efficiency is calculated through the ratio of household carbon emissions to energy consumption to reflect the CO_2_ emissions per unit of energy consumption.


$$Y=\sum_{i=1}^{8}X_{i}\times C_{i}$$ (2)

In Eq (2), *Y* denotes the total energy consumption and carbon emission of household consumption, *X*_*i*_ denotes the energy intensity and carbon intensity of consumption of category *i*, and *C*_*i*_ denotes the expenditure of household consumption that shows category *i*. Household energy efficiency is the result of total household carbon emissions/total energy consumption.

#### 3.3.2 Household digital transformation (DIGIT)

Household digital transformation (DIGIT) is the core explanatory variable in this study. We construct a comprehensive measure of household digitalization [53] that captures its potential impact on energy efficiency and emissions reduction. Our indicator design is based on previous studies [54, 55] and aligns with the theoretical framework in our literature review. To measure household digital transformation, we consider two main aspects: accessibility and usage of digital technologies. These aspects are chosen to represent the overall digitalization level of households, as suggested by Yin et al. [54] and Luan et al. [55]. Under these two aspects, we include four specific indicators:

1. Smartphone usage (accessibility): This shows households’ access to mobile internet, which can help energy-efficient behaviors [24].
2. Computer ownership (accessibility): This represents households’ ability for digital interactions, potentially enabling better energy management [25].
3. Mobile payment adoption (usage): This shows households’ use of digital financial services, which may reduce carbon emissions [56].
4. Online shopping behavior (usage): This captures households’ e-commerce activity, which can change consumption patterns and possibly reduce energy use [30].

These indicators relate to our literature review on how digital transformation can affect household energy efficiency. Smartphones and computers relate to the "integration of the Internet with power-consuming devices" [25, 26]. Mobile payments and online shopping reflect the "changed level and structure of household consumption" [24].

Data for these indicators come from the China Household Finance Survey (CHFS), as shown in Table 2. We use Principal Component Analysis (PCA) to create a single measure. The KMO value of 0.758 (>0.5) shows our indicators are suitable for PCA.

**Table 2:** Comprehensive indicator system for digital transformation of households.

Standard level	Source of questionnaire questions	Index Interpretation
Whether to use a smartphone	What type of cell phone are you currently using?	Yes = 1; Otherwise = 0
Whether to use a computer	Which of the following types of durable goods does your household currently own, and what is the value of each of them?	Yes = 1; Otherwise = 0
Whether to use mobile payment	At present, does your household have third−party payment accounts, such as Alipay, WeChat Pay, Jingdong Netbanking Wallet, Baidu Wallet, etc.?	Yes = 1; Otherwise = 0
Whether to purchase online	How much did your family spend in total on online shopping last year?	Yes = 1; Otherwise = 0

#### 3.3.3 Control variables

This work sets the following control variables on the basis of existing literature references [57–59] and data availability. The first set includes household characteristics variables, including per capita household income, household size, percentage of elderly population, percentage of minor population, and home ownership. The second set includes household head characteristics variables, including the household head’s household type, age, gender, political profile, education level, marital status, and health status. Table 3 demonstrates the description of the assignment of the variables used in this paper.

**Table 3:** Definition of variables.

Variable type	Variable name	Metrics	Description of the assignment
Implicit variable	Home energy efficiency (EFFI)	Household carbon emissions/household energy consumption	Logarithm of the actual value
Independent variable	Household digital transformation (DIGIT)	Household use of smartphones, computers, mobile payments, online shopping	Principal component analysis method
Control variables	Age	Year of birth	2019 − Year of birth and squared
	Gender	Gender	Male = 1; female = 0
	Political profile (Political)	Political appearance	CCP member = 1; other = 0
	Educational attainment (Educ)	Educational attainment	Never attended school = 1; Elementary school = 2; Junior high school = 3; High school/vocational high school/technical school/secondary school = 4; College = 5; Bachelor’s degree and above = 6
	Marital status (Marital)	Current marital status	In marital status = 1;
	Hukou type (Hukou)	Current Hukou type	Not in marriage = 0
	Per capita income (lnincome)	Per capita household income based on total annual household income	Agriculture = 1; Other = 0
	Household size (H−Size)	Total household size	
	Ratio of older persons (OldRto)	Ratio of persons aged 65 or above to the number of persons in the household	
	Ratio of Minors (MinRto)	Number of persons under 18 years old as a percentage of household size	
	Housing property rights (H−Rights)	Property rights of the house in which he/she lives	1 = owned by household members; 2 = rented; 3 = free residence
	Health status (Health)	Health status of the head of household	1 = very good; 2 = good; 3 = fair; 4 = bad; 5 = very bad;

The results in Table 4 show that the levels of energy efficiency across households have large disparities. It can be seen that in terms of household energy efficiency, the gap is nearly 25 times between the maximum value and the minimum value. The mean value of household energy efficiency is 1.579, indicating that most households’ energy efficiency are at a relatively low level. In the variable of digital transformation of households, some households have not experienced digital transformation, and large differences exist in digital transformation between individual households. The minimum value of household digital transformation is -2.237, the maximum value is 1.207, indicating that most households’ digital transformation level are not high. Other variables are within a reasonable range overall.

**Table 4:** Descriptive statistics.

Variable	Obs	Mean	Std.Dev.	Min	Max
EFFI	12485	1.579	0.32	1.254	32.445
DIGIT	12485	0	1.249	−2.237	1.207
Hukou	12485	0.499	0.5	0	1
Age	12485	54.448	13.797	18	99
Age2	12485	3154.962	1513.467	324	9801
Political	12485	0.206	0.404	0	1
Gender	12485	0.736	0.441	0	1
Educ	12485	3.394	1.316	1	6
Marital	12485	0.873	0.333	0	1
lnincome	12485	9.875	1.366	−2.436	14.779
Health	12485	0.438	0.496	0	1
H−Rights	12485	1.203	0.524	1	3
H−Size	12485	3.174	1.539	1	15
OldRto	12485	0.239	0.373	0	1
MinRto	12485	0.143	0.185	0	1

## 4 Empirical results

### 4.1 Baseline results

To verify the impact of household digital transformation on household energy efficiency, we conducted a regression of Eq (1). The results are shown in Table 5. First, Table 5(1) represents the examination of the impact of household digital transformation on household energy efficiency without the inclusion of control variables and without controlling for province fixed effects. It shows that household digital transformation can significantly contribute to household energy efficiency. Next, Table 5(2) further controls for province fixed effects and adds control variables for household head characteristics. Its regression results are still significant. Finally, Table 5(3) adds household−level control variables, and the regression results again show that household digital transformation can significantly contribute to household energy efficiency. We argue that households have undergone a digital transformation and that the digital economy coming into the home requires the introduction of many digital technologies, which themselves require a large amount of energy consumption to support them. However, households have undergone digital transformation that encourages energy efficiency. Thus, Hypothesis 1 is validated.

**Table 5:** Baseline regression results.

	(1)	(2)	(3)
	EFFI	EFFI	EFFI
DIGIT	−0.071***	−0.048***	−0.033***
	(0.002)	(0.003)	(0.003)
Hukou		0.051***	0.052***
		(0.007)	(0.007)
Age		0.000	0.001
		(0.001)	(0.002)
Age2		0.000	−0.000
		(0.000)	(0.000)
Gender		−0.006	−0.002
		(0.007)	(0.007)
Political		−0.006	−0.004
		(0.007)	(0.007)
Educ		−0.013***	−0.013***
		(0.003)	(0.003)
Marriage		−0.068***	−0.044***
		(0.009)	(0.009)
Health		−0.004	−0.000
		(0.006)	(0.006)
lnincome			−0.023***
			(0.002)
H−Rights			−0.019***
			(0.005)
H−Size			−0.024***
			(0.002)
OldRto			0.019
			(0.012)
MinRto			−0.027
			(0.021)
Const	1.579***	1.577***	1.899***
	(0.003)	(0.043)	(0.053)
Province	NO	YES	YES
R^2^	0.076	0.103	0.120
N	12485	12485	12485

Note

*p < 0.1

**p < 0.05

***p < 0.01, values in parentheses are standard errors (same below).

### 4.2 Endogeneity analysis

To overcome endogeneity, this study uses the instrumental variable method for estimation. We draw on Zhang et al. [38] and Zhang and Zhong [60]. The instrumental variables we have selected are the distance of the household’s region from the provincial capital city and the municipal digital financial inclusion index. Theoretically, the instrumental variable clearly possesses relevance to household digital transformation. The provincial capital is usually the economic center of a province. The closer to the provincial capital city is, the better the digital development should be. This correlation is closely related to the level of Internet access and application. In such case, more households tend to achieve digital transformation. Regions with better digital inclusion indices are also highly likely to be regions with higher Internet penetration, which is generally the basis for household digital transformation [61]. Hence, the correlation and exogeneity principles of instrumental variables are satisfied.

We first examined the validity of the instrumental variables. Table 6 reports the results. This outcome shows that the two sets of instrumental variables we have selected are highly justified. The results of the second−stage regression show that after overcoming the potential endogeneity problem, household digital transformation and household energy efficiency still maintain a significant correlation. This outcome does not change essentially from the results of the benchmark regression. Thus, it further corroborates the conclusions obtained from the benchmark regression and confirms the robustness of the estimation results.

**Table 6:** Instrumental variable tests.

	(1)	(2)	(3)	(4)
	First Stage	Second Stage	First Stage	Second Stage
	DIGIT	EFFI	DIGIT	EFFI
IV1 (Digital Inclusive Finance Index)	0.004*** (5.489)			
IV2 (Distance from the capital city of the province to which it belongs)			−0.001*** (−5.114)	
DIGITL		−0.194***		−0.249***
		(−2.678)		(−2.892)
Weak IV test (F−statistic)	30.13		26.15	
Anderson canon. corr. LM statistic		30.161		26.194
(P-val)		0.0000		0.0000
Cragg-Donald Wald F statistic		30.127		26.150
Stock-Yogo critical value (10%)		16.38		16.38
Sargan statistic (overidentification test of all instruments)			0.403	
Chi-sq(1) P-val			0.5256	
Province	YES	YES	YES	YES
Control	YES	YES	YES	YES
N	11473	11473	10127	10127
R^2^	0.533		0.529	

### 4.3 Robustness tests

We employed four robustness checks, as shown in Table 7. They are as follows: (1) calculating household energy efficiency per capita as the explanatory variable, (2) shrinking household energy efficiency by 1% to mitigate extreme values, (3) excluding samples from first-tier and new first-tier cities, and (4) removing samples with negative household digital transformation values. After the robustness test using the above methods, the promotion effect of household digital transformation on household energy efficiency remains valid.

**Table 7:** Robustness tests.

	(1)	(2)	(3)	(4)
	Substitution of variables	Shrinkage treatment	Selection of subsamples	Selection of subsamples
	EFFIAVG	EFFI_w	EFFI	EFFI
DIGIT	−0.033***	−0.033***	−0.033***	−0.039***
	(0.003)	(0.001)	(0.004)	(0.003)
Const	1.709***	1.741***	2.049***	1.703***
	(0.059)	(0.019)	(0.078)	(0.020)
R^2^	0.510	0.444	0.097	0.251
Province	YES	YES	YES	YES
Control	YES	YES	YES	YES
N	12485	12485	8555	6745

### 4.4 Mechanism testing

#### 4.4.1 Mediating effects

According to the previous theoretical analysis, household digital transformation can improve household energy efficiency by increasing household income level, which in turn changes consumption structure and lifestyle. If this path is valid, the level of household income will change the impact of digital transformation on energy efficiency. Specifically: (1) When household income level is higher, the additional income growth brought by digital transformation may be used to purchase higher quality and more environmentally friendly products and services. It is expected that the effect of digital transformation on improving energy efficiency will be more significant. (2) As income levels rise, households’ demand for environmental quality will also increase, and they are more likely to adopt energy-saving and environmentally friendly lifestyles. It is expected that the effect of digital transformation on improving energy efficiency through income will be more obvious. Based on these expectations, we construct the following model:

$$EFFI_{i}=\alpha +\beta_{1}DIGIT_{i}+\beta_{2}Lnincome_{i}+\beta_{3}LnDIGIT_{i}\times Lnincome_{i}+\sum \gamma_{j}X_{i}+\varepsilon_{i}$$ (3)

In the first step of the regression for (1) in Table 8, the estimated coefficient of household digital transformation for household energy efficiency is significantly negative, thus indicating that household digital transformation can improve household energy efficiency. In the second step of (2) of Table 8, the estimated coefficient of household digital transformation for household per capita income is significantly positive, thereby indicating that household digital transformation facilitates household per capita income. The third step adds household per capita income to the baseline regression, and the result remains significant. This outcome represents a more significant mediating effect of household per capita income of about 13.99%. To verify the mediation mechanism more rigorously, we use the bias−corrected nonparametric percentile bootstrap method to analyze the mediation effect. Table 9 shows that the confidence interval (95% Conf. Interval) for the pattern household digital transformation → household per capita income → household energy effect is (−0.0081,−0.0038) without 0. This outcome suggests that household per capita income is an important mechanism by which household digital transformation improves household energy efficiency. Hence, Hypothesis 3a is supported. In addition, we simultaneously use the Sobel method for testing, which is consistent with the above results. From the validation results, household digital transformation significantly increases the level of household per capita income, which optimizes household energy efficiency. Household digital transformation means that family members have acquired additional digital skills and digital literacy. Digital skills and digital literacy can expand family income−generating channels, thus providing wide possibilities for families to obtain employment opportunities and optimize asset allocation, which is conducive to raising the income level and gradually improving the living standard. Improved quality of life does not entail increased carbon emissions from household consumption [62]. Rather, it pertains to choosing a higher quality and greener quality of life. Such option changes traditional consumption patterns and reduces the probability of selecting an energy−intensive lifestyle, thus changing the consumption structure, which subsequently affects household energy efficiency.

**Table 8:** Mechanism testing.

	(1)	(2)	(3)	(4)
	EFFI	lnincome	EFFI	EFFI
DIGIT	−0.039***	0.230***	−0.033***	−0.073***
	(0.003)	(0.012)	(0.003)	(0.007)
lnincome			−0.023***	
			(0.002)	
Educaverage				−0.036***
				(0.005)
DIGIT*Educaverage				0.017***
				(0.002)
Const	1.654***	10.481***	1.899***	1.904***
	(0.048)	(0.181)	(0.053)	(0.054)
R^2^	0.113	0.300	0.120	0.127
Province	YES	YES	YES	YES
Control	YES	YES	YES	YES
N	12485	12485	12485	12485

**Table 9:** Mechanism testing: Bootstrap results.

	Coef.	Std. Err.	95% Conf. Interval		Percentage of Mediating effects.
lnincom(dir_eff)	−0.0332	0.0020	−0.0367	−0.0290	13.99%
lnincom(ind_eff)	−0.0054	0.0011	−0.0081	−0.0038	

Note: Bootstrap sample size is 500.

#### 4.4.2 Moderating effects

Based on the previous theoretical analysis, the impact of household digital transformation on household energy efficiency may be moderated by the household education level. If this moderating effect exists, the education level of the household would alter the strength of the influence of digital transformation on energy efficiency. Specifically: (1) When the household education level is higher, family members may be more capable of understanding and applying digital technologies, thereby more effectively utilizing the energy-saving opportunities brought by digital transformation. It is expected that the enhancing effect of digital transformation on energy efficiency would be more significant. (2) Households with higher education levels may have stronger awareness and concern about environmental issues, and are more inclined to apply digital technologies to energy conservation and emission reduction. It is anticipated that the effect of digital transformation on improving energy efficiency would be more pronounced. However, there is also an opposite possibility: (3) Households with higher education levels may have higher income and consumption capacity, gaining access to more energy-intensive products, which may offset some of the energy-saving effects brought by digitalization.

As shown in Table 8 column (4), the educational attainment per household presents significance in the modeling of energy efficiency, i.e., a moderating effect exists. Therefore, Hypothesis 3b is supported. Household education per person weakens the impact of household digital transformation on energy efficiency. Balaguer and Cantavella [63] revealed that access to more education increases people’s income and purchasing power, which in turn provides access to energy-intensive technologies. Therefore, in this case, education may lead to an increase in CO_2_. Notably, the moderating effect of education per household is complex. The use of digital technologies is more prevalent among the educated population, and we believe that education and environmental sustainability remain closely linked. Environmental education is indispensable in expanding access to education. In the future, environmental education should be prominently integrated into basic and higher education programs to help students form the right attitude toward energy conservation.

### 4.5 Further analysis

This part will further discuss the following two aspects. Firstly, it discusses the exact distribution pattern of the impact of household digital transformation on households with different levels of household energy efficiency. The second is pertains to whether variations exist in the impact of household digital transformation on household energy efficiency among different types of household energy efficiency and different household base characteristics.

#### 4.5.1 Quantile regression

We used ordinary least squares to conduct the benchmark regression, which is also a commonly used method in finding empirical evidence. This method focuses on the mean value. While quantile regression focuses on the impact on the dependent variable at different quantiles, the regression parameters can change with different distribution points of the dependent variable, which is more suitable for achieving a detailed and comprehensive analysis of the regression relationship between phenomena. Households with different levels of consumption expenditures produce varying energy consumption and carbon emissions, which ultimately lead to differences in energy efficiency. Therefore, this study five quartiles for analysis, namely, 10%, 25%, 50%, 75%, and 90%. As shown in Table 10. The coefficients of household digital transformation are always significant regardless of whether the values are under the low quartile or under the middle and high quartiles. This trend indicates that household digital transformation improves household energy efficiency, which is consistent with the previous findings. Differences exist in the ultimate energy efficiency resulting from households with different levels of consumption. However, the contribution of household digital transformation is the same and always encourages the optimization of household energy efficiency.

**Table 10:** Quantile regression.

	(1)	(2)	(3)	(4)	(5)
	10%	25%	50%	75%	90%
DIGIT	−0.019***	−0.023***	−0.028***	−0.038***	−0.053***
	(0.002)	(0.001)	(0.001)	(0.001)	(0.003)
Const	1.523***	1.605***	1.748***	1.945***	2.160***
	(0.029)	(0.020)	(0.021)	(0.025)	(0.049)
Province	YES	YES	YES	YES	YES
Control	YES	YES	YES	YES	YES
N	12485	12485	12485	12485	12485

#### 4.5.2 Analysis of heterogeneity across consumption categories

The energy consumption and carbon emissions generated by various types of consumption in households have several differences. Moreover, the resulting energy efficiencies are inconsistent. As such, we inquire whether heterogeneity exists in the household energy efficiency effects of household digital transformation on various types of consumption. To determine this impact, we have constructed a consumption type heterogeneity analysis to explore the effects of household digital transformation on household energy efficiency for each of the eight types of consumption. As shown in Table 11, household digital transformation has various effects on the energy efficiency generated by different household consumption types. The regression results show that household digital transformation can effectively improve the energy efficiency of food, housing, and other consumption. In addition, differences exist in the effects of household digital transformation on the energy efficiency of different household consumption types, with the intensity and significance of the effects varying greatly.

**Table 11:** Heterogeneity test: Different consumption types.

	(1)	(2)	(3)	(4)	(5)	(6)	(7)	(8)
	Food	Clothing	Resident	Equipment	Medical	Travel	Education	Other
DIGIT	−0.000***	0.346***	−0.002**	0.013**	0.022	0.035***	0.475***	−0.249***
	(0.000)	(0.028)	(0.001)	(0.006)	(0.024)	(0.008)	(0.034)	(0.039)
Const	8.903***	5.886***	8.719***	8.937***	7.509***	7.487***	5.427***	7.222***
	(0.000)	(0.472)	(0.014)	(0.095)	(0.401)	(0.144)	(0.576)	(0.669)
R^2^	0.028	0.101	0.006	0.012	0.035	0.043	0.166	0.074
Province	YES	YES	YES	YES	YES	YES	YES	YES
Control	YES	YES	YES	YES	YES	YES	YES	YES
N	12485	12485	12485	12485	12485	12485	12485	12485

#### 4.5.3 Heterogeneity of households

The consumption expenditure needs of different types of households vary. Therefore, we analyze the heterogeneous impact of household digital transformation on household energy efficiency from the perspectives of households with or without the elderly and households with or without minors. In addition, judging the difference in coefficients between groups only by comparing the size of the coefficients between groups is too arbitrary. Moreover, this comparison can easily deviate from the real situation. Therefore, the significance of the difference in coefficients between groups must be tested. In this study, the Suest method is used to examine the difference in coefficients between groups. The results are shown in Table 12.

**Table 12:** Heterogeneity test: Different household types.

	(1)	(2)	(3)	(4)
	without the elderly	with the elderly	without minors	with minors
DIGIT	−0.038***	−0.023***	−0.026***	−0.032***
	(0.002)	(0.007)	(0.005)	(0.002)
Suest	chi2(1) = 8.18***		chi2(1) = 1.79	
	Prob > chi = 0.0042		Prob > chi2 = 0.1811	
Const	1.736***	2.260***	1.934***	1.752***
	(0.029)	(0.174)	(0.092)	(0.028)
R^2^	0.384	0.087	0.100	0.348
Province	YES	YES	YES	YES
Control	YES	YES	YES	YES
N	7864	4621	7327	5158

Firstly, regressions are grouped by households with or without the elderly. Table 12 columns (1) and (2) regress households with older people over 65 years of age as “with older people” households and other households as “without older people” households. In the regression of households with or without elderly people, the digital transformation of households has a significant positive impact on the energy efficiency of both groups of households. The difference in the coefficients between the groups passed the significance test at the 1% statistical level. This outcome indicates that the positive effect of home digital transformation on energy efficiency is greater for households with the elderly.

Secondly, returns are grouped according to the households with or without minors. Table 12 columns (3) and (4) are regressed on households with minors under the age of 18 as “with minors” and other households as “without minors”. In the regressions for households with and without minors, the difference in coefficients between groups does not pass the test, thus indicating that the difference between the two groups is not significant.

## 5 Conclusion and discussion

We have empirically examined the impact of household digital transformation on household energy efficiency on the basis of CHFS data. The results of the study show that the following. ① Household digital transformation can improve household energy efficiency. ② This transformation enhances household energy efficiency under the low quartile as well as under the middle and high quartiles. ③ At the same time, household digital transformation has different effects on the energy efficiency generated by various household consumption types. Nevertheless, this transformation enhances the energy efficiency generated by food, housing, and other consumption. ④ In addition, the positive effect of the digital transformation of households on the energy efficiency of households with elderly people is greater than those without elderly people. ⑤ Moreover, per capita household income and per capita household education are important mechanisms that influence the relationship between household digital transformation and household energy efficiency. Our findings confirm that household digital transformation is an important factor in improving energy efficiency and helps to alleviate the dilemma between digital technology development and environmental protection.

Our findings extend the existing literature on household energy efficiency and digital transformation in several ways. While prior studies have mainly focused on the macro-level impacts of digitalization [17, 18, 64], our research provides micro-level evidence from Chinese households. Moreover, unlike studies that only examine the overall effect of digital economy [19], we decompose household digital transformation into multiple dimensions and analyze their heterogeneous impacts.

Our results reveal novel insights into the relationship between digital transformation and energy efficiency at the household level. Contrary to some studies suggesting digitalization may increase energy consumption [22], we find that household digital transformation can improve energy efficiency when properly measured. This highlights the importance of considering both energy use and carbon emissions when evaluating efficiency.

Future studies could explore the long-term dynamics between household digital transformation and energy efficiency, as well as potential non-linear relationships. Cross-country comparisons would also be valuable to test the generalizability of our findings beyond the Chinese context.
